# LncRNA-mediated DNA methylation: an emerging mechanism in cancer and beyond

**DOI:** 10.1186/s13046-022-02319-z

**Published:** 2022-03-15

**Authors:** Wanxu Huang, Hua Li, Qingsong Yu, Wei Xiao, Dan Ohtan Wang

**Affiliations:** 1 Wuya College of Innovation, Shenyang Pharmaceutical University, Shenyang, 110016 Liaoning China; 2 The Fifth Affiliated Hospital of Guangzhou Medical University, Guangzhou, 510700 Guangdong China; 3 State Key Laboratory of New-Tech for Chinese Medicine Pharmaceutical Process, Lianyungang, 222001 Jiangsu China; 4 Department of Laboratory Medicine, The First Affiliated Hospital of China Medical University, Shenyang, 110001 Liaoning China; 5 Jiangsu Kanion Pharmaceutical CO. LTD, Lianyungang, 222001 Jiangsu China; 6 Center for Biosystems Dynamics Research, RIKEN, 2-2-3 Minatojima-minamimachi, Chuo-ku, Kobe, Hyogo 650-0047 Japan

**Keywords:** lncRNA, DNA methylation, Non-coding RNA, DNMT, TET, Cancer

## Abstract

DNA methylation is one of the most important epigenetic mechanisms to regulate gene expression, which is highly dynamic during development and specifically maintained in somatic cells. Aberrant DNA methylation patterns are strongly associated with human diseases including cancer. How are the cell-specific DNA methylation patterns established or disturbed is a pivotal question in developmental biology and cancer epigenetics. Currently, compelling evidence has emerged that long non-coding RNA (lncRNA) mediates DNA methylation in both physiological and pathological conditions. In this review, we provide an overview of the current understanding of lncRNA-mediated DNA methylation, with emphasis on the roles of this mechanism in cancer, which to the best of our knowledge, has not been systematically summarized. In addition, we also discuss the potential clinical applications of this mechanism in RNA-targeting drug development.

## Background

DNA methylation is the methyl modification on the fifth carbon of cytosines (5-methylcytosine, 5mC) typically found in the context of symmetrical CpG dinucleotides in mammals [1, 2]. It is estimated that 70–80% of CpG sites in the mammalian genome are methylated [3], excluding specific regions called CpG islands (CGIs). CGIs are CpG-rich sequences of about 1 kilo-base (kb) in length that mostly exist in gene promoters [4]. Approximately 60% of human gene promoters contain CGIs [5].

DNA methylation is established by DNA methyltransferases (DNMTs). In the simplified but widely accepted ‘division of labor’ model, it is proposed that DNMT3A and DNMT3B are essential for de novo DNA methylation, while DNMT1 is for methylation maintenance during DNA replication [6]. Ten-eleven translocation (TET) family of enzymes (TET1, TET2, and TET3) oppose the actions of the DNMT family by oxidation of 5mC, followed by replication-dependent dilution or thymine DNA glycosylase (TDG)-dependent base excision repair, leading to active DNA demethylation [7–9].

Genome-scale analysis revealed distinct DNA methylation patterns across different cell types, developmental stages, and in response to different stimuli [3, 10, 11]. Aberrant DNA methylation pattern is associated with diseases, including cancer [12–15]. In cancer cells, whereas the general DNA methylation levels are reduced, the CGIs are hypermethylated in a cancer-specific manner [16, 17]. These observations raised a fundamental question: how does the cell type-specific DNA methylation pattern established across the genome? It is well-demonstrated that histone modification and chromosome remodeling [18], as well as transcriptional factors, play key roles in the regulation of DNA methylation genome-wide and in site-specific manner [19–22]. Studies in recent years have accumulated compelling evidence to suggest that long non-coding RNA (lncRNA) is another important regulator of DNA methylation, especially in cancer.

While less than 2% of the human genome encodes proteins, nearly three-quarters can be actively transcribed into non-coding RNAs [23], amongst the ones typically with length more than 200 nucleotides are cataloged as lncRNAs. According to a current statistical analysis, there are more than 173,112 annotated lncRNAs transcribed from 96,411 genomic loci [24]. It is demonstrated that lncRNAs play versatile roles in development and diseases including cancer [25–27]. In the nucleus, lncRNAs regulate chromatin remodeling and transcription; In the cytoplasm, lncRNAs regulate translation and mRNA turnover (reviewed in ref. [27]). There is accumulating evidence up to date showing that lncRNAs mediate DNA methylation via multiple manners, thereby regulating target gene expression in diverse physiological and pathological processes. In this review, we summarize our current understanding of lncRNA-mediated DNA methylation, with emphasis on the functions of this mechanism in cancer. The future direction and potential clinical application are also discussed.

### LncRNAs recruit DNA methyltransferases

More than a decade ago, it was discovered that lncRNAs transcribed from the promoter of rRNA genes (rDNA) regulate DNA methylation and transcription of rDNA [28]. Later, it was demonstrated that this kind of lncRNA interacts with rDNA promoter and forms a DNA: RNA triplex, which is recognized by DNMT3B to epigenetically regulate rDNA expression [29, 30]. Although it is still unclear if this is a common model nowadays, a variety of lncRNAs have been reported to recruit DNMTs and regulate target gene expression, playing key roles in mesoderm commitment [31], muscle regeneration [32, 33], neural differentiation [34], adipogenesis [35], mental disorder [36], cardiovascular diseases [37–40], osteoarthritis [41], as well as types of cancer (Table 1).

**Table 1:** LncRNAs mediate DNA methylation in cancer

lncRNA	Role	Factor	Target	Function	Cancer	Ref
TINCR	Recruit	DNMT1	miR-503-5p	Regulate EGFR expression	BC	[42]
MROS-1	Recruit	DNMT3A	PRUNE2	Nodal metastases	OC	[43]
HOTAIR	Recruit	DNMT1	PTEN	Cell proliferation, invasion and migration	CML	[44]
LINC00887	Recruit	DNMT1	CA9	Suppress oncogenic CA9	TSCC	[45]
LINC00472	Recruit	DNMTs	MCM6	Inhibited tumor growth and metastasis	TNBC	[46]
LINC01270	Recruit	DNMTs	GSTP1	Promote tumorigenesis and drug resistance	EC	[47]
DLX6-AS1	Recruit	DNMT1	LARGE	Promotes Lymph Node Metastasis	PCa	[48]
HOTAIR	Recruit	DNMTs	MTHFR	chemoresistance	EC	[49]
ADAMTS9-AS2	Recruit	DNMT1/3	CDH3	Inhibits proliferation, invasion, and migration	EC	[50]
IRAIN	Recruit	DNMT1/3	VEGFA	Suppresses tumor growth	RC	[51]
PVT1	Recruit	DNMT1	miR-18b-5p	Promotes proliferation	GBC	[52]
DLX6-AS1	Recruit	DNMTs	CADM1	Maintenance of cancer stem cells	HCC	[53]
BZRAP1-AS1	Recruit	DNMT3b	THBS1	Promotes angiogenesis	HCC	[54]
KCNQ1OT1	Recruit	DNMT1	Kcnq1	Promotes chemoresistance	OSA	[55]
PYCARD-AS1	Recruit	DNMT1, G9a	PYCARD	Regulates apoptosis	BC	[56]
MIR210HG	Recruit	DNMT1	CACNA2D2	Promotes proliferation and invasion	NSCLC	[57]
HAGLR	Recruit	DNMT1	E2F1	Suppresses tumor growth	LUAD	[58]
DACOR1	Recruit	DNMT1	Genome-wide		CRC	[59, 60]
LINC00628	Recruit	DNMTs	LAMA3	Promotes tumorigenesis and drug resistance	LUAD	[61]
PVT1	Recruit	DNMT1	BNIP3	Promotes cell proliferation	GC	[62]
HOTAIR	Recruit	DNMT3B	HOXA5	Promotes cell proliferation	AML	[63]
MALAT1			Mitochondrial DNA	Control metabolic Reprogramming	HCC	[64]
HOTAIR	Upregulate	DNMT3b	PTEN	Doxorubicin resistance	AML	[65]
RP11-159K7.2	Upregulate	DNMT3A		Promotes cell growth and invasion	LSCC	[66]
GAS5	Down-regulate	DNMTs	miR-424	Suppresses multiple malignant phenotypes	Glioma	[67]
lnc-OIP5-AS1	Upregulate	DNMT1	pre-miR-218–1	Promote cell motility and proliferation	KS	[68]
Linc-GALH	Ubiquitinate	DNMT1	Gankyrin	Promotes metastasis	HCC	[69]
LUCAT1	Inhibits ubiquitination	DNMT1	tumor-suppressor genes	Promotes tumor formation and metastasis	ESCC	[70]
HOTAIR	Upregulate (via EZH2)	DNMTs	miR-122	Activate Cyclin G1 and promote tumorigenicity	HCC	[71]
HOTAIR	Upregulate	DNMT1/3B	HOXA1	Multidrug resistance	SCLC	[72]
H19	Upregulate	TET3	MED12	Promotes cell proliferation	UL	[73]
DBCCR1-003	Sequestrate	DNMT1	DBCCR1	Inhibits cell growth	BCa	[74]
TTTY15	Sequestrate	DNMT3A	TBX4	Suppresses metastasis	NSCLC	[75]
HOTAIRM1	Sequestrate	G9a/EZH2/ DNMTs	HOXA1	Promotes tumor growth and invasion	GBM	[76]
91H	Repel	DNMTs	H19/IGF2 locus	Promotes tumorigenesis	BC	[77]
HOTAIR	Recruit (via EZH2)		HOXA1	Multidrug resistance	SCLC	[78]
SNHG3	Recruit (via EZH2)		MED18	Promotes cell migration and invasion	GC	[79]
HOXB13-AS1	Recruit (via EZH2)	DNMT3B	HOXB13	Promotes cell proliferation	Glioma	[80]
Lnc-LALC	Recruit (via EZH2)	DNMTs	LZTS1	Liver metastasis	CRC	[81]
HOTAIR	Recruit (via EZH2)	DNMT1	miR-454-3p	Promotes tumor growth	CS	[82]
GIHCG	Recruit (via EZH2)	DNMT1	miR-200b/a/429	Promotes tumor growth and metastasis	HCC	[83]
LINC00630	Restrict (via EZH2)	DNMT3B	BEX1	Suppresses cell apoptosis and promotes radio-resistance	CRC	[84]
Lnc34a	Recruit (via PHB2)	DNMT3A	miR-34a	Promotes cell proliferation	CRC	[85]
H19	Inhibit (via inhibiting SAHH)	DNMT3b	Beclin1	Induces autophagy activation and tamoxifen resistance	BC	[86]
LINC00662	Regulate	MAT1A/ SAHH		Activates SAM-dependent oncogenes	HCC	[87]
SNHG6	Regulate (via miRNAs)	MAT1A, MAT2A	Genome-wide		HCC	[88]
H19	Inhibit (via inhibiting SAHH)	DNMTs	LINE-1	Benzo [*a*]pyrene (BaP) carcinogenesis	Lung cancer	[89]
MAGI2-AS3	Recruit	TET2	LRIG1	Inhibits the self-renewal of leukaemic stem cells	AML	[90]
SSTR5-AS1	Recruit	TET1	E-cadherin	Inhibits tumor progression and metastasis	LSCC	[91]
SATB2-AS1	Recruit (via GADD45A)	TETs	SATB2	Inhibits cell metastasis and regulates immune response	CRC	[92]

*Abbreviations:* *BC* Breast cancer, *OC* Oral cancer, *CML* Chronic myeloid leukemia, *TSCC* Tongue squamous cell carcinoma, *TNBC* Triple-negative breast cancer, *EC* Esophageal cancer, *PCa* Prostate cancer, *RC* Renal carcinoma, *GBC* Gallbladder cancer, *HCC* Hepatocellular carcinoma, *OSA* Osteosarcoma, *NSCLC* Non-small cell lung cancer, *LUAD* Lung adenocarcinoma, *CRC* Colorectal cancer, *GC* Gastric cancer, *AML* Acute myeloid leukemia, *LSCC* Laryngeal squamous cell carcinoma, *KS* Kaposi’s sarcoma, *ESCC* Esophageal squamous cell carcinoma, *SCLC* small-cell lung cancer, *UL* Uterine leiomyomas, *GBM* Glioblastoma multiforme, *CS* Chondrosarcoma

**Fig. 1:**
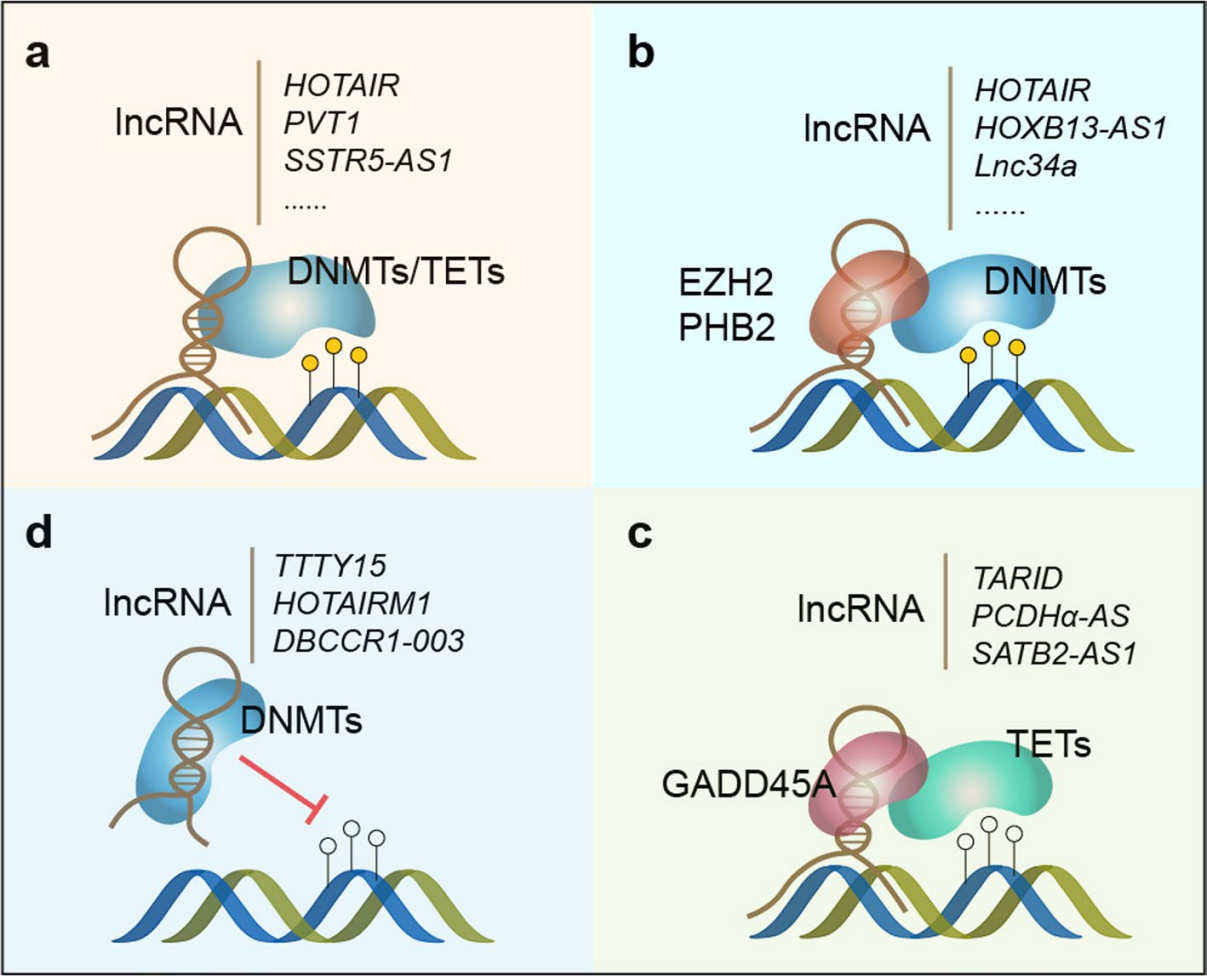
LncRNA interacts with DNMTs/TETs. **a.** LncRNAs directly recruit DNMTs/ TETs. **b.** LncRNAs indirectly recruit DNMTs via EZH2/PHB2. **c.** LncRNAs indirectly recruit TETs via GADD45A. **d.** LncRNAs sequestrate DNMTs

Using an optimized RIP-seq method, Merry et al. identified 148 lncRNAs interacting with DNMT1 in colon cancer cells [59], and the following investigation showed that one of these lncRNAs, *DACOR1*, could recruit DNMT1 and reprogram genome-wide DNA methylation [60]. Currently, a growing number of studies suggest that lncRNA might recruit DNMTs directly to specific targets (Fig. 1a), including both protein-coding genes [43, 44, 46, 47, 49–51, 55, 57, 58, 62, 63] and non-coding genes such as miRNA [42, 52, 93]. For instance, in esophageal cancer (EC), lncRNA *ADAMTS9-AS2* was reported to recruit DNMT1/3 to *CDH3* promoter, inhibiting the cancer cell proliferation, invasion, and migration [50]. Two other lncRNAs, *HOTAIR* and *LINC01270* might recruit DNMTs to the promoters of *MTHFR* and *GSTP1* respectively, leading to chemoresistance in EC [47, 49]. In lung adenocarcinoma (LUAD), lncRNA *HAGLR* was identified as a tumor suppressor by recruiting DNMT1 to the promoter of *E2F1* to inhibit tumor growth [58]. A recent study revealed a more complex scenario, in which the authors identified two novel variants of lncRNA *LINC00887*, and showed that the short form variant suppressed *Carbonic Anhydrase IX* (*CA9*) by recruiting DNMT1 to its promoter, while the long-form variant activated *CA9*'s transcription via interacting with HIF1α [45]. The two variants were supposed to differentially respond to hypoxia and oppositely control the progression of tongue squamous carcinoma [45].

**Fig. 2:**
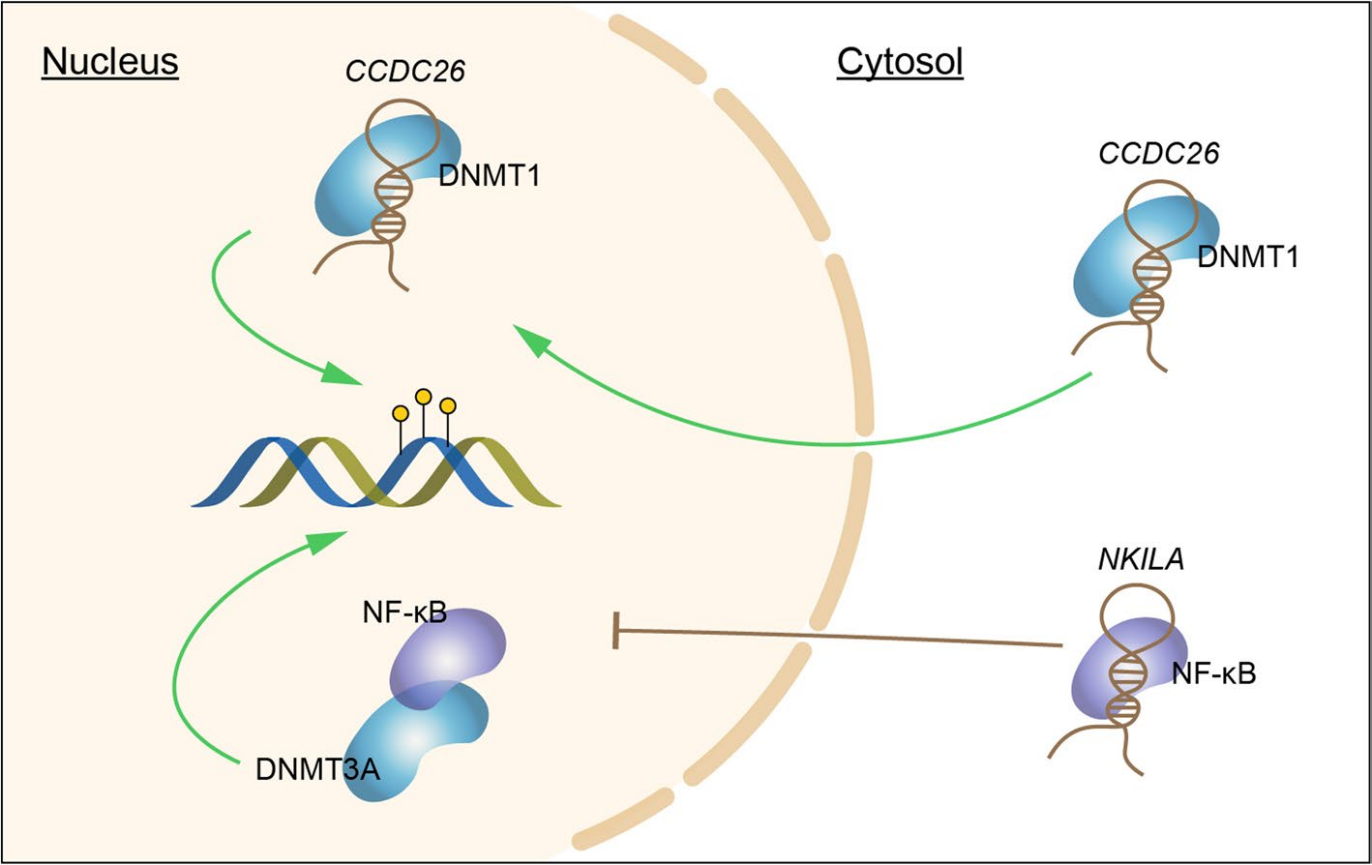
LncRNAs regulate DNMT activity via nucleocytoplasmic shuttling. LncRNA *CCDC26* interacts with DNMT1 and promotes its localization from the cytosol to the nucleus. LncRNA *NKILA* sequesters NF-κB in the cytoplasm, which hinders NF-κB recruitment of DNMT3A in the nucleus

Meanwhile, several groups also proposed that lncRNAs could recruit DNMT indirectly through the mediation of other factors (Fig. 1b). It was previously proposed that the polycomb group (PcG) protein EZH2 (Enhancer of Zeste homolog 2) interacts with DNMT and associates with DNMT activity [94]. Studies in recent years demonstrated in diverse cancers that lncRNAs might regulate DNA methylation of target genes via association with EZH2, promoting tumor growth [80, 82], metastasis [79, 81, 83] and radio-resistance [84]. Alternatively, EZH2 might regulate DNA methylation by the formation of H3K27me3 histone modification [78], while the molecular mechanism involved in H3K27me3-induced DNA methylation is unclear. Apart from histone modifier EZH2, two transcriptional regulators, NF-κB and PHB2 were also reported to interact with DNMT3A [85, 95]. LncRNA *NKILA* was identified as a suppressor of NF-κB by sequestering NF-κB in cytoplasm [96]. Upon proinflammatory stimuli, NF-κB is released from the sequestration and translocated into the nucleus (Fig. 2). DNMT3A is then recruited to the promoter of *KLF4* by NF-κB, repressing *KLF4* transcription by DNA methylation [95]. Another study by Wang et al*.* reported a lncRNA called *Lnc34a*, which could interact with Prohibitin 2 (PHB2) and then recruit DNMT3A to *miR-34a* promoter, silencing *miR-34a* expression and promoting colorectal cancer growth [85]. PHB2 is a multi-functional protein that can shuttle between nucleus and mitochondria [97]. Interestingly, the nuclear-encoded lncRNA *MALAT1* was recently discovered to be transported into mitochondria and to regulate the methylation status of mitochondrial DNA in hepatocellular carcinoma [64], yet the detailed mechanism is unclear.

While most of the reported function of lncRNA recruitment of DNMT is to target DNMT to specific genomic sites or regions, recent work from Jones et al*.* proposed a different model, in which the lncRNA *CCDC26* specifically interacts with DNMT1 and promote its localization from the cytosol to nucleus (Fig. 2), while removal of *CCDC26* leads to genome-wide hypomethylation, increasing double-stranded DNA breaks and inducing cell death [98]. More investigation is needed to confirm if the interaction is direct and to reveal the detailed mechanisms.

### LncRNAs recruit TET enzymes

TET (Ten-eleven Translocation)-mediated 5mC oxidation is responsible for the active erasure of DNA methylation [99]. Studies from recent years have revealed that a subset of lncRNAs has the potential to interact with TETs and regulate DNA methylation (Table 1).

In some cases, lncRNA directly interacts with TETs and recruits them to specific targets (Fig. 1a). It was demonstrated that lncRNA *Oplr16* binds to the *Oct4* promoter, orchestrating the promoter-enhancer loops and then interacts with TET2 by the 3' region of *Oplr16 *[100]. Similarly, Du et al*.* identified two motifs in lncRNA *Platr10* that interact with *Oct4* promoter and TET1 respectively, thus inducing TET1- mediated DNA demethylation at specific site [101]. A research by Zhou et al*.* suggested that lncRNA *TETILA* regulates TET2 subcellular localization and enzymatic activity by binding to the DSBH (double-stranded β-helix) domain of TET2 [102]. In acute myeloid leukemia, lncRNA *MAGI2-AS3* recruits TET2 to *LRIG1* promoter, inducing up-regulation of *LRIG1* and inhibition of leukemic stem cell self-renewal [90]. Interestingly, using RNA reverse transcription-associated trap sequencing (RAT-seq) approach to profile genome-wide interaction targets for lncRNAs in mice, a recent study reported that lncRNA *Peblr20* recruits TET2 to the enhancer of *Pou5F1* and activates the enhancer-transcribed RNAs [103]. Whether a similar mechanism exists in humans especially in cancer development remains uninvestigated.

There is also evidence supporting an indirect model (Fig. 1c), in which lncRNAs recruit TET via GADD45A. It was first reported by Arab et al*.* that an antisense lncRNA from *TCF21* gene locus termed *TARID* might recruit GADD45A (growth arrest and DNA-damage-inducible, alpha), and GADD45A then recruits TET to the promoter of its partner gene and induce its activation by DNA demethylation [104]. In the following work, the authors further showed that *TARID* forms an R-loop at the *TCF21* promoter to recruit GADD45A [105]. It was speculated that lncRNA *PCDHα-AS* might function in a similar mechanism to recruit TET3 via GADD45A, driving stochastic promoter choice to establish a neuronal surface identity code for circuit assembly [106]. In colorectal cancer (CRC), lncRNA *SATB2-AS1* directly recruits WDR5 and GADD45A, promoting SATB2 transcription by histone modification, as well as DNA demethylation [92], which inhibits cell metastasis and regulates the immune response in CRC. Recently, a database was created, with a comprehensive list of R-loops and their respective regulatory proteins [107], which might serve as a useful resource to identify novel lncRNAs with the potential to recruit GADD45A via formation of R-loops.

### LncRNAs repel/ sequestrate DNA methyltransferases

While most of the current reports suggest the DNMT-recruiting role of lncRNAs, some lncRNAs are also shown to repel or sequestrate DNMT to negatively regulate DNA methylation (Fig. 1d and Table 1).

It was first reported by Di Ruscio et al*.* that a lncRNA arising from the *CEBPA* gene locus binds to DNMT1 and prevents *CEBPA* promoter methylation [108]. The lncRNA *DBCCR1-003* was reported to function similarly to suppress *DBCCR1* promoter methylation by sequestrating DNMT1 and eventually to inhibit cell growth in bladder cancer [74]. In non-small cell lung cancer, lncRNA *TTTY15* interacts with DNMT3A and inhibits the binding of DNMT3A to *TBX4* promoter, while the lower expression level of *TTTY15* is associated with tumor metastasis [75]. In glioblastoma, lncRNA *HOTAIRM1* was suggested to interact with several epigenetic factors including DNMT1/3A/3B to sequester them away from *HOXA1* promoter [76]. In breast cancer, it was discovered that lncRNA *91H*, which is transcribed from the antisense orientation of *H19*, promotes oncogenesis by masking methylation site on the H19 promoter, inducing the oncogenic *H19* overexpression [77].

### LncRNAs control SAM/ SAH level to regulate DNMT activity

**Fig. 3:**
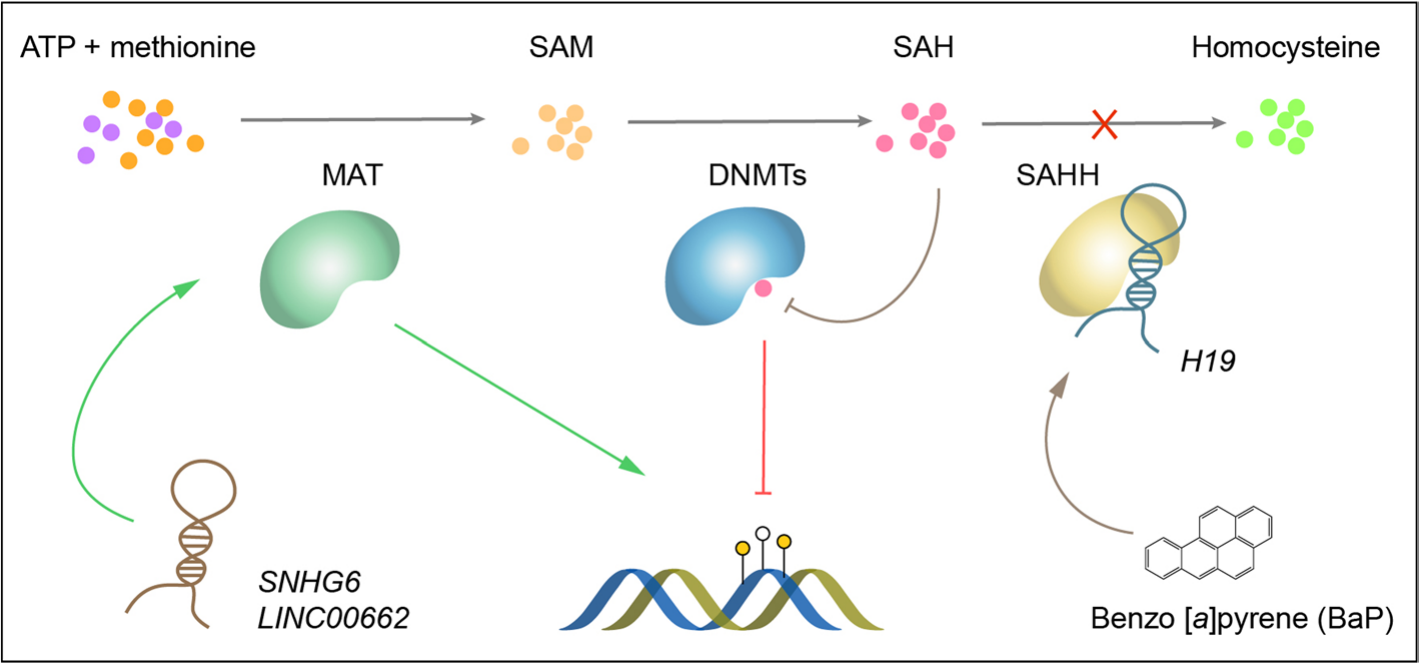
LncRNAs control SAM/SAH level to regulate DNMT activity. S-adenosylhomocysteine (SAM) is biosynthesized by MAT (methionine adenosyltransferase) and converted to SAH (S-adenosylhomocysteine) by DNMTs. SAH is also a strong feedback inhibitor of DNMTs and it can be cleaved by S-adenosylhomocysteine hydrolase (SAHH). LncRNAs control SAM/SAH level by interacting with MAT or SAHH, and carcinogens such as benzo [*a*]pyrene (BaP) might enhance the interaction

DNMT catalyzes transmethylation reactions using S-adenosylmethionine (SAM) as the methyl group donor, yielding S-adenosylhomocysteine (SAH) as a by-product, which is also a strong feedback inhibitor of DNMT [6]. In mammals, SAM is biosynthesized by methionine adenosyltransferase (MAT) from ATP and methionine [109], while SAH is reversibly cleaved into adenosine and homocysteine by S-adenosylhomocysteine hydrolase (SAHH, also known as AdoHcy hydrolase, AHCY), which is essential to prevent accumulation of SAH [109], thereby relieving its inhibition to DNMT (Fig. 3).

It was proposed that lncRNA *H19* binds to and inhibits SAHH, leading to genome-wide methylation changes at numerous gene loci [110]. Afterward, this mechanism was verified in embryonic hematopoietic stem cell development [111], odontogenic differentiation [112], metabolic abnormality [113] and neurodegenerative diseases [114]. In breast cancer, it was demonstrated that *H19* inhibits SAHH, resulting in the accumulation of SAH, which restricts DNMT3B from methylating *Beclin1* promoter and inducing the upregulation of *Beclin1* and subsequently initiates autophagy, contributing to tamoxifen resistance [86]. Interestingly, the interaction of *H19* and SAHH might be enhanced by Benzo [*a*]pyrene (BaP), which is a potent carcinogen, especially in lung cancer [89].

Other than the SAH level regulated by SAHH, the SAM level regulated by MAT is another factor affecting DNMT activity (Fig. 3). MAT has several homologs and isoenzymes, among which, MAT1A is mainly expressed in adulthood, serving as a marker for the normal differentiated liver. While MAT2A is a marker for rapid liver growth and dedifferentiation, which is transcriptionally induced in hepatocellular carcinoma (HCC) [109]. It was reported that the oncogenic lncRNA *SNHG6* upregulates *MAT2A* expression as a competitive endogenous RNA (ceRNA) to sponge miR-1297, while down-regulates *MAT1A* translation by suppressing nucleocytoplasmic shuttling of *MAT1A* mRNA, thereby causing genome-wide hypomethylation and promoting HCC [88]. Recently, the same group of investigators identified a novel lncRNA named *LINC00662* that was shown to decay *MAT1A* mRNA by RNA–RNA interactions and degrades SAHH protein by ubiquitination [87]. These studies revealed a pathway regulating the level of SAM/SAH to further control DNMT activity, with broad functions in cancer and other diseases.

### LncRNAs regulate the expression of DNMTs/ TETs

**Fig. 4:**
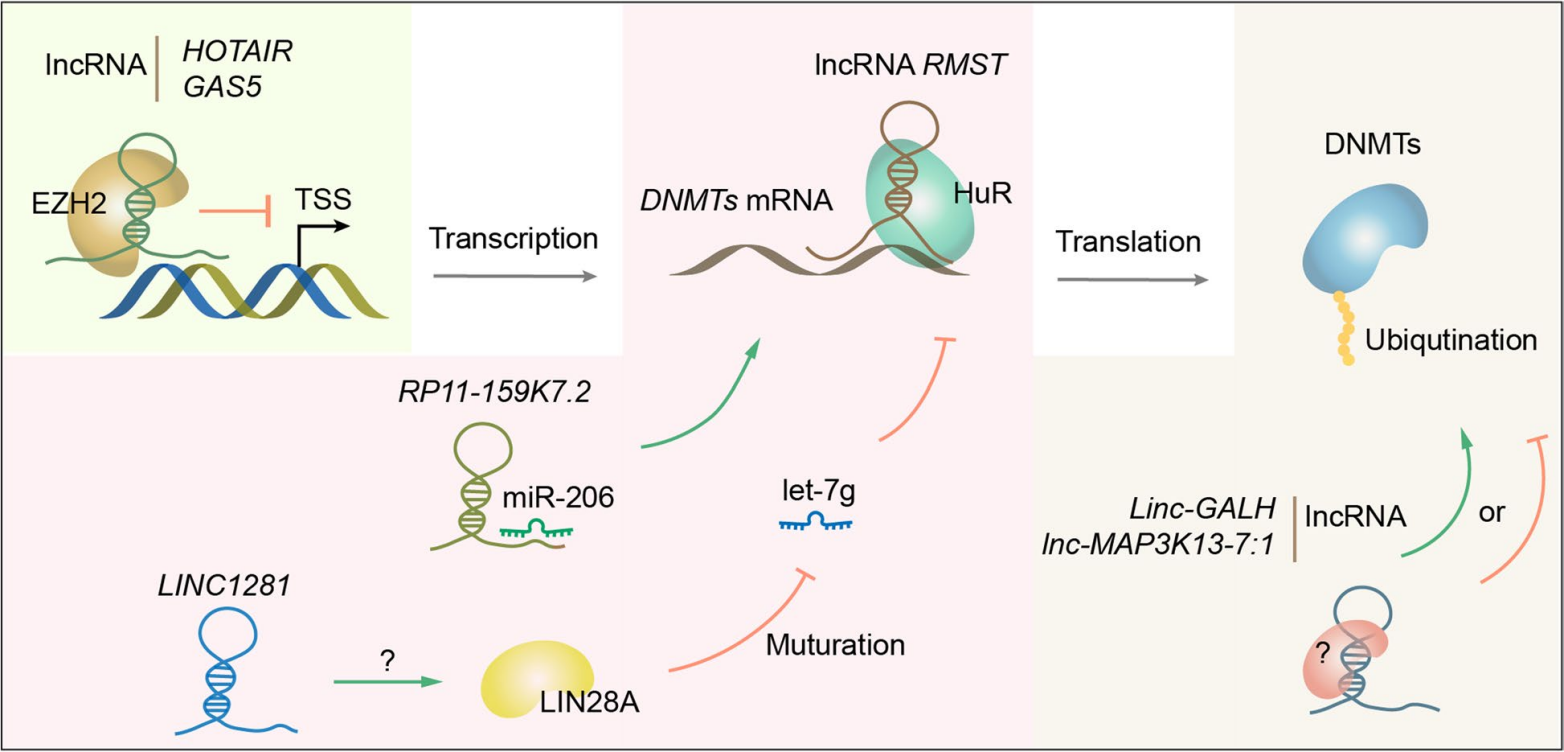
LncRNAs regulate the expression of DNMTs/ TETs at diverse levels. Firstly, lncRNAs might regulate the transcription of *DNMTs* via interaction with EZH2 to form repressive chromatin; Secondly, lncRNAs can stabilize *DNMTs* mRNA by recruiting HuR or as a miRNA sponge; Thirdly, lncRNAs regulate DNMT on the protein level by promoting or inhibiting its ubiquitination

There is compelling evidence showing that lncRNAs control the expression of DNMTs and TETs at diverse levels to regulate DNA methylation (Table 1 and Fig. 4). It was reported that lncRNAs promote or suppress DNMT expression, playing key roles in osteogenesis [115], macrophage polarization [116], as well as cell invasion in Kaposi's sarcoma [68] and chemoresistance in small cell lung cancer [72] and acute myeloid leukemia [65]. Several molecular mechanisms of lncRNA’s regulatory effect on DNMTs or TETs have been elucidated (Fig. 4).

The first mechanism is to regulate the transcription, as demonstrated in malignant glioma, where lncRNA *GAS5* directly interacts with EZH2 and stimulates the formation of polycomb repressive complex 2 (PRC2), thereby transcriptionally suppressing *DNMT *[67]. There is also a report suggesting that EZH2 is recruited by lncRNA *HOTAIR* to upregulate DNMT, while the mechanism is unclear [71].

The second mechanism is to regulate the stability of *DNMT* mRNA, where lncRNA functions as a mediator to upregulating DNMT by interaction with the stabilizing factor HuR [117], or as a ceRNA to sponge specific miRNA, thereby upregulating DNMT [66]. The latter mechanism was also discovered in TET regulation, where estradiol and progesterone upregulate lncRNA *H19* to suppress miRNA Let-7 and stabilize *TET3* mRNA, activating key fibroid-promoting genes in uterine leiomyomas [73]. LncRNA might also exert this effect via a more indirect manner, as demonstrated for *LINC1281*, which stabilizes the expression of *Let-7* miRNA, thus down-regulating its targets DNMT3A/B [118].

The third mechanism is to regulate DNMT at the protein level. Current studies mainly focus on protein degradation by ubiquitination (Fig. 4). It was reported by several groups that lncRNAs serve as a protein-binding scaffold and induce ubiquitin-mediated DNMT protein degradation, epigenetically regulating target gene expression in obesity-mediated beta cell dysfunction [119], polycystic ovary syndrome [120] and hepatocellular carcinoma (HCC) [69]. The detailed mechanism involving the role of lncRNA in DNMT ubiquitination is largely unknown and warrant more deep investigation. In esophageal squamous cell carcinoma, a distinct model was proposed, in which, the lncRNA *LUCAT1* binds DNMT1 to protect it from ubiquitination, while *LUCAT1* knock-down promotes ubiquitination of DNMT1 through UHRF1 (Ubiquitin-Like PHD and RING Finger Domain-Containing Protein 1) [70]. However, it is well established that UHRF1 deposits dual mono- ubiquitination on the H3 histone tail and PCNA-associated factor 15 (PAF15) for direct DNMT1 recruitment and DNA methylation maintenance [121–123], while its roles in the mediation of DNMT1 ubiquitination need further validation and investigation.

## Conclusions and discussions

Studies in recent years have revealed the multi-faceted role of lncRNA in regulating DNA methylation. Firstly, lncRNAs can recruit or repel DNA modifiers (DNMTs/ TETs) to specific gene targets (Fig. 1; Fig. 2); Secondly, lncRNAs can regulate DNMT activity by controlling the level of DNMT cofactor SAM/ SAH (Fig. 3); Lastly, lncRNAs can regulate the expression of DNMTs/ TETs per se at multiple levels (Fig. 4). All these mechanisms have been investigated in development and disease, with emphasized roles in cancer.

While most of the studies focused on the DNA methylation of the gene promoters, there is also a recent report highlighting the gene-body methylation mediated by a lncRNA by recruiting DNMT3A, which facilitates transcription of *CTSG* in dermatomyositic myoideum [124]. Whether this mechanism exists in cancer needs further investigation.

Although this review mainly discussed the lncRNA function in mediating DNA methylation, another two issues should be noted. The first is that lncRNAs are in turn regulated targets of DNA methylation [125–128]; The second is that lncRNAs also mediate other epigenetic alterations such as histone modification and chromosome remodeling [129–136]. These issues provide an additional layer of gene expression regulation to form complex crosstalk between lncRNA, transcriptional factors, and various epigenetic modifications. More elaborate investigations are warranted to reveal the common mechanisms.

### Perspectives

**Fig. 5:**
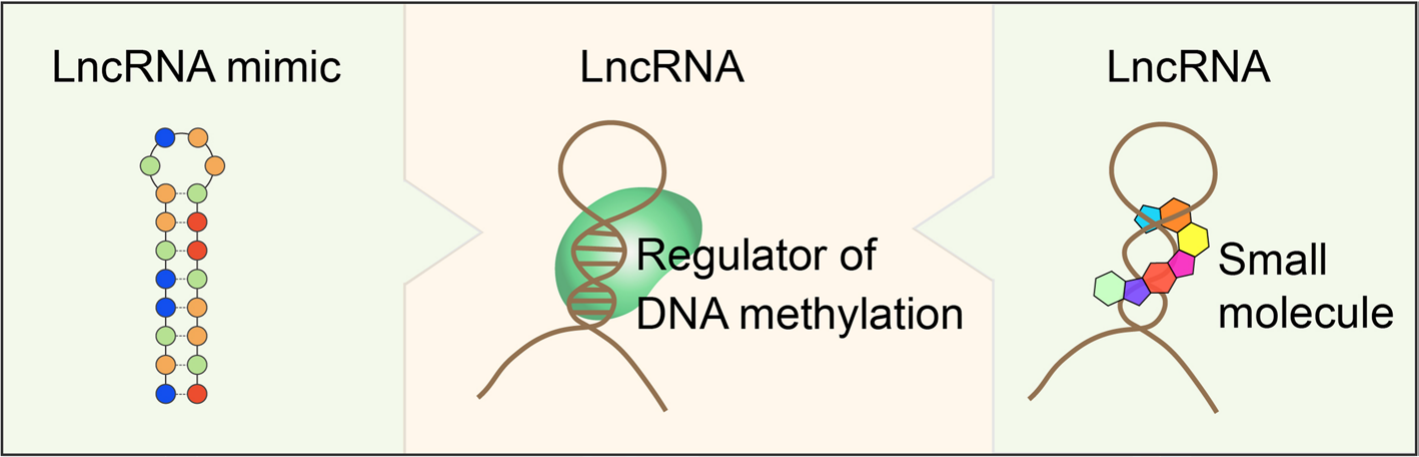
Potential therapeutic strategies for lncRNA targeting based on lncRNA- mediated DNA methylation. The middle panel shows the interaction between lncRNA and DNA methylation regulators, which could be specifically interrupted by small molecular compounds (right panel). Alternatively, the regulators could also be modulated by lncRNA mimics (left panel)

The emerging roles of lncRNAs in cancer through the mediation of DNA methylation suggest novel applications in drug development. While there are currently no drugs targeting lncRNA based exactly on this mechanism, relevant studies shed light on this field (Fig. 5).

One direction is to design lncRNA mimics to regulate the activity of their target proteins, which was recently applied in treating a rare disease of phenylketonuria, where a lncRNA *HULC* was identified to interact with phenylalanine hydroxylase (PAH) and to modulate the enzymatic activities of PAH. In their work, the authors constructed a lncRNA mimic that rescues PAH enzymatic activity in *HULC*-deficient cells and mouse models, which showed the therapeutic potential for phenylketonuria [137].

Another direction is to design small molecules directly targeting lncRNA-protein interactions [138–141]. Based on the structural insight of the interaction between lncRNA *HOTAIR* and EZH2, Ren et al*.* conducted a high-throughput virtual screening and identified a compound that selectively interrupts the lncRNA-protein interaction and inhibits cancer cell invasion and migration [142].

Owing to the fast progress of RNA structural biology and screening technologies, as well as the in-depth mechanistic studies and drug delivery technologies, it is reasonable to expect that RNA-targeting will emerge as a growing therapeutic strategy for human disorders, especially cancer.

## Data Availability

Not applicable.

## Abbreviations

lncRNA: Long non-coding RNA
CGIs: CpG islands
DNMTs: DNA methyltransferases
TET: Ten-eleven translocation
TDG: Thymine DNA glycosylase
PcG: Polycomb group
PRC2: Polycomb repressive complex 2
EZH2: Enhancer of Zeste homolog 2
GADD45A: Growth arrest and DNA-damage-inducible alpha
SAM: S-adenosylmethionine
SAH: S-adenosylhomocysteine
MAT: Methionine adenosyltransferase
SAHH: S-adenosylhomocysteine hydrolase
ceRNA: Competitive endogenous RNA
BC: Breast cancer
OC: Oral cancer
CML: Chronic myeloid leukemia
TSCC: Tongue squamous cell carcinoma
TNBC: Triple-negative breast cancer
EC: Esophageal cancer
PCa: Prostate cancer
RC: Renal carcinoma
GBC: Gallbladder cancer
HCC: Hepatocellular carcinoma
OSA: Osteosarcoma
NSCLC: Non-small cell lung cancer
LUAD: Lung adenocarcinoma
CRC: Colorectal cancer
GC: Gastric cancer
AML: Acute myeloid leukemia
LSCC: Laryngeal squamous cell carcinoma
KS: Kaposi’s sarcoma
ESCC: Esophageal squamous cell carcinoma
SCLC: Small-cell lung cancer
UL: Uterine leiomyomas
GBM: Glioblastoma multiforme
CS: Chondrosarcoma
