# Correction: LncRNA-mediated DNA methylation: an emerging mechanism in cancer and beyond

**DOI:** 10.1186/s13046-022-02468-1

**Published:** 2022-08-27

**Authors:** Wanxu Huang, Hua Li, Qingsong Yu, Wei Xiao, Dan Ohtan Wang

**Affiliations:** 1 Wuya College of Innovation, Shenyang Pharmaceutical University, Shenyang, 110016 Liaoning China; 2 The Fifth Affiliated Hospital of Guangzhou Medical University, Guangzhou, 510700 Guangdong China; 3 State Key Laboratory of New-Tech for Chinese Medicine Pharmaceutical Process, Lianyungang, 222001 Jiangsu China; 4 Department of Laboratory Medicine, The First Affiliated Hospital of China Medical University, Shenyang, 110001 Liaoning China; 5 Jiangsu Kanion Pharmaceutical CO. LTD, Lianyungang, 222001 Jiangsu China; 6 Center for Biosystems Dynamics Research, RIKEN, 2-2-3 Minatojima-minamimachi, Chuo-ku, Kobe, Hyogo 650-0047 Japan

**Correction: J Exp Clin Cancer Res 41, 100 (2022)**

**https://doi.org/10.1186/s13046-022-02319-z**

Following publication of the original article [1], an error was identified in five references; specifically:

Reference 48. Zhao Z, Liang S, Sun F. LncRNA DLX6-AS1 Promotes Malignant Phenotype and Lymph Node Metastasis in Prostate Cancer by Inducing LARGE Methylation. Front Oncol. 2020;10:1172.

Reference 53: Wu DM, Zheng ZH, Zhang YB, Fan SH, Zhang ZF, Wang YJ, et al. Downregulated lncRNA DLX6-AS1 inhibits tumorigenesis through STAT3 signaling pathway by suppressing CADM1 promoter methylation in liver cancer stem cells. J Exp Clin Cancer Res. 2019;38:237.

Reference 55. Qi X, Yu XJ, Wang XM, Song TN, Zhang J, Guo XZ, et al. Knockdown of KCNQ1OT1 Suppresses Cell Invasion and Sensitizes Osteosarcoma Cells to CDDP by Upregulating DNMT1-Mediated Kcnq1 Expression. Mol Ther Nucleic Acids. 2019;17:804–18.

Reference 61: Xu SF, Zheng Y, Zhang L, Wang P, Niu CM, Wu T, et al. Long Non-coding RNA LINC00628 Interacts Epigenetically with the LAMA3 Promoter and Contributes to Lung Adenocarcinoma. Mol Ther Nucleic Acids. 2019;18:166–82.

Reference 63: Wang SL, Huang Y, Su R, Yu YY. Silencing long non-coding RNA HOTAIR exerts anti-oncogenic effect on human acute myeloid leukemia via demethylation of HOXA5 by inhibiting Dnmt3b. Cancer Cell Int. 2019;19:114.

The reference citations and their respective bibliographic details have been deleted. The correction does not have any effect on the results or conclusions of the paper. The original article has been corrected.
